# A radical opposition in body weight control

**DOI:** 10.1002/emmm.201303094

**Published:** 2013-08-05

**Authors:** Michael Ristow, Christian Wolfrum

**Affiliations:** 1 Laboratory for Energy MetabolismETH Zurich, Zurich, Switzerland; 2 Laboratory for Translational Nutrition BiologyETH Zurich, Zurich, Switzerland

**Keywords:** adipogenesis, adipose tissue, obesity, reactive oxygen species

Reactive oxygen species (ROS) are generally considered to be detrimental for health because of their association with a wide variety of diseases including, but not limited to type 2 diabetes, cardiovascular complications and cancer; as such, they have been considered to be undesirable by-products of aerobic metabolism. This concept has led to the widespread use of antioxidant food supplements to promote health as well as to prevent and even treat certain disease types. In recent years, however our view on ROS and antioxidants has shifted, as more and more evidence is accumulating that ROS are actually fundamental signalling molecules in many cellular processes and that excessive ROS scavenging may have damaging side effects.

The study by Chang et al (2013) in this issue provides additional evidence for this notion as it aims to unravel the role of ROS signalling in adipogenesis. It is well accepted that ROS signalling controls fat cell formation *in vitro*; the pathways that govern this process, however, are still incompletely understood. In the current study, the authors show that glutathione peroxidase 7 (GPx7 or NPGx) is an essential sensor of ROS in pre-adipocytes and controls their differentiation into mature adipocytes. NPGx belongs to the oxidative stress sensor/transducer family that currently encompasses eight members. Interestingly, NPGx is quite selectively expressed in pre-adipocytes contained within the stromal vascular fraction of adipose tissue and is downregulated upon adipogenesis. As previously recognised (Lee et al 2009), the authors demonstrate that ROS induction drives adipocyte formation. The NPGx-mediated changes in ROS levels lead to the activation of the protein kinase A (PKA)/CEBPβ axis in addition to regulating mitotic clonal expansion in pre-adipocytes, a step which is thought to be a critical determinant of adipocyte formation. Thus, the authors add an important building block to the expanding field of ROS mediated signalling in cell differentiation.

Several factors have been implicated in ROS mediated induction of adipogenesis, including NADPH oxidase (Nox), nitric oxide synthase (Nos) (Kanda et al 2011) as well as mitochondrial metabolism, with a special contribution from complex I and III (Carriere et al 2003). Nevertheless, which of these pathways is responsible for generating the ROS that are necessary to induce adipogenesis, is incompletely understood at the moment. Similarly, which pathway(s) is/are responsible for the transmission of ROS signals remains unclear. Possible suggested candidates are members of the protein tyrosine phosphatase class as well as AMP-kinase signalling, as well as the transcription factor CEBPβ because of the oxidative sensing capabilities of the active dimer (Kim et al 2007), however information in the context of adipogenesis has been lacking until now. Interestingly, PPARγ, the master regulator of adipogenesis, is also sensitive to the cellular redox state although the underlying mechanism of this regulation is still incompletely understood (Zhang et al 2006). The report by Chang et al (2013) now demonstrates that CEBPβ plays a major role in mediating ROS signals. They demonstrate, in a series of elegant experiments, that ROS control CEBPβ activity at the post-transcriptional level through PKA signalling, possibly promoting clonal expansion and thereby adipogenesis. Interestingly, the authors also show an upregulation of PPARγ expression in response to increased ROS mediated by NGPx knockdown which is only evident late in differentiation, therefore other factors might contribute in this signalling pathway. In conclusion, several lines of evidence suggest that ROS induce adipogenesis by controlling cell proliferation and maturation.

…ROS control CEBPβ activity at the post-transcriptional level through PKA signalling, possibly promoting clonal expansion and thereby adipogenesis

Although obesity and oxidative stress are clearly correlated, it still remains unclear whether this relationship is causal and if so, which factor is responsible for driving the other. Furthermore, long-term oxidative stress is considered to be at the root of several metabolic diseases. In this study, the authors show that global ablation of NPGx leads to increased adiposity and concomitant insulin resistance. Treatment of mice with ROS scavengers such as *N*-acetylcysteine (NAC) can rescue the observed phenotype. In addition, the authors describe a SNP upstream of the NPGx gene, which is associated with adiposity in different cohorts. A simple conclusion that can be drawn from these two findings is that increased ROS levels lead to an induction of adipogenesis and subsequent insulin resistance. However, and as it often happens, there are problems with this line of thinking. On the one hand adiposity will not be caused by increased adipogenesis alone. An imbalance in energy intake or expenditure is a prerequisite for such a phenotype. Indeed, the study shows that NPGx mice have decreased energy expenditure possibly due to a decrease in ambulatory activity. This defect is ablated when mice are treated with NAC, suggesting that ROS is responsible for certain behaviour paradigms. On the other hand, it is well known that increased PPARγ activity and adipogenesis in conjunction with metabolic overload can lead to adiposity but is protective with regard to the development of insulin sensitivity (Spiegelman 1998). Also in the present study, the authors report only a slight and not significant increase in adipocyte numbers, which is an indication that other pathways are modulated by ROS and NAC and contribute to the observed phenotype. One possibility could be that ROS produced by adipocytes or their precursors leads to effects in other tissues. In this respect, it is worth recalling that more than 30 years ago, H_2_O_2_ was shown to mimic the effects of insulin stimulation, *albeit* in islet cells (Lipkin et al 1983). Since prolonged insulin signalling can cause insulin resistance one might speculate that such a mechanism might underlie the observed phenotype.

…ROS may have diverse or even opposing roles in different compartments of the body and therefore, in principle, the use of ROS-reducing pharmaceutical or supplemental interventions to tackle human obesity is unlikely to be successful

Interestingly, and in contrast to the current findings on NPGx, absence of GPx1, another member of the NPG family, which likewise is a sensor and transducer of ROS signalling leads to a protection from obesity and the associated metabolic disorders (Loh et al 2009). Conversely, overexpression of GPx1 promotes obesity and insulin resistance in mice (McClung et al 2004), and supplementation with antioxidants may promote insulin resistance in humans (Ristow et al 2009). Unlike NPGx, GPx1 is expressed in most tissues and supplementation similarly affects most tissues regarding their antioxidant defense capacity. Moreover, it is unclear whether GPx1 also controls adipogenesis or whether the effect is mediated through other tissues, as GPx1 is much more widely expressed. To address this, it will be of utmost importance to develop animal model systems in which expression can be directly modulated in the pre-adipocyte, especially since the current data imply that ROS signalling in pre-adipocytes may differ from that in other insulin sensitive tissues, also given the predominant expression of NPGx in adipocytes and their precursors. The findings therefore suggest that ROS may have diverse or even opposing roles in different compartments of the body and therefore, in principle, the use of ROS-reducing pharmaceutical or supplemental interventions to tackle human obesity is unlikely to be successful.

The authors declare that they have no conflict of interest.
